# Membrane guanylyl cyclase complexes shape the photoresponses of retinal rods and cones

**DOI:** 10.3389/fnmol.2014.00045

**Published:** 2014-06-02

**Authors:** Xiao-Hong Wen, Alexander M Dizhoor, Clint L Makino

**Affiliations:** ^1^Department of Ophthalmology, Massachusetts Eye and Ear Infirmary and Harvard Medical SchoolBoston, MA, USA; ^2^Department of Basic Sciences Research and Pennsylvania College of Optometry, Salus UniversityElkins Park, PA, USA

**Keywords:** guanylate cyclase, guanylyl cyclase activating protein, Ca^2+^ feedback, Ca^2+^-binding protein, phototransduction, rod and cone photoreceptors, knockout mouse, neuronal calcium sensors

## Abstract

In vertebrate rods and cones, photon capture by rhodopsin leads to the destruction of cyclic GMP (cGMP) and the subsequent closure of cyclic nucleotide gated ion channels in the outer segment plasma membrane. Replenishment of cGMP and reopening of the channels limit the growth of the photon response and are requisite for its recovery. In different vertebrate retinas, there may be as many as four types of membrane guanylyl cyclases (GCs) for cGMP synthesis. Ten neuronal Ca^2+^ sensor proteins could potentially modulate their activities. The mouse is proving to be an effective model for characterizing the roles of individual components because its relative simplicity can be reduced further by genetic engineering. There are two types of GC activating proteins (GCAPs) and two types of GCs in mouse rods, whereas cones express one type of GCAP and one type of GC. Mutant mouse rods and cones bereft of both GCAPs have large, long lasting photon responses. Thus, GCAPs normally mediate negative feedback tied to the light-induced decline in intracellular Ca^2+^ that accelerates GC activity to curtail the growth and duration of the photon response. Rods from other mutant mice that express a single GCAP type reveal how the two GCAPs normally work together as a team. Because of its lower Ca^2+^ affinity, GCAP1 is the first responder that senses the initial decrease in Ca^2+^ following photon absorption and acts to limit response amplitude. GCAP2, with a higher Ca^2+^ affinity, is recruited later during the course of the photon response as Ca^2+^ levels continue to decline further. The main role of GCAP2 is to provide for a timely response recovery and it is particularly important after exposure to very bright light. The multiplicity of GC isozymes and GCAP homologs in the retinas of other vertebrates confers greater flexibility in shaping the photon responses in order to tune visual sensitivity, dynamic range and frequency response.

## INTRODUCTION

Unlike most neurons, retinal rods and cones are partially depolarized while at rest (in darkness). Cation channels in the open state permit an influx of Na^+^ and also some Ca^2+^ that is termed the “dark” current. When rods and cones receive light, photon capture by visual pigment within the outer segment is amplified by the subsequent activation of many transducin (G-protein) molecules, each of which stimulate phosphodiesterase (PDE) enzymatic activity. Cyclic GMP (cGMP) hydrolysis leads to closure of cyclic nucleotide-gated (CNG) channels thereby blocking the dark current. The ensuing hyperpolarization of membrane potential spreads through the photoreceptor to reduce neurotransmitter release at the synaptic terminal. To recover quickly from stimulation by light, cGMP, the second messenger that links photon capture to CNG channel opening, must be restored (for reviews on phototransduction, see Luo et al., 2008; Wensel, 2008; Gross and Wensel, 2011; Korenbrot, 2012).

Membrane guanylyl cyclases (GCs) catalyze the synthesis of cGMP. As opposed to other membrane GCs, those of photoreceptors do not respond to extracellular ligands, but instead are subject to regulation by Ca^2+^-binding, EF-hand bearing, GC activating protein (GCAP) subunits (reviewed in Sharma and Duda, 2012; Koch and Dell’Orco, 2013; Lim et al., 2014). GCAP suppresses synthesis of cGMP by GC in darkness, when intracellular Ca^2+^ is relatively high. After light closes the CNG channels and blocks Ca^2+^ influx, continued extrusion by Na^+^/K^+^, Ca^2+^ exchangers lowers intracellular Ca^2+^. Ca^2+^ dissociates from GCAP allowing it to bind Mg^2+^ instead, whereupon it stimulates GC activity.

**FIGURE 1:**
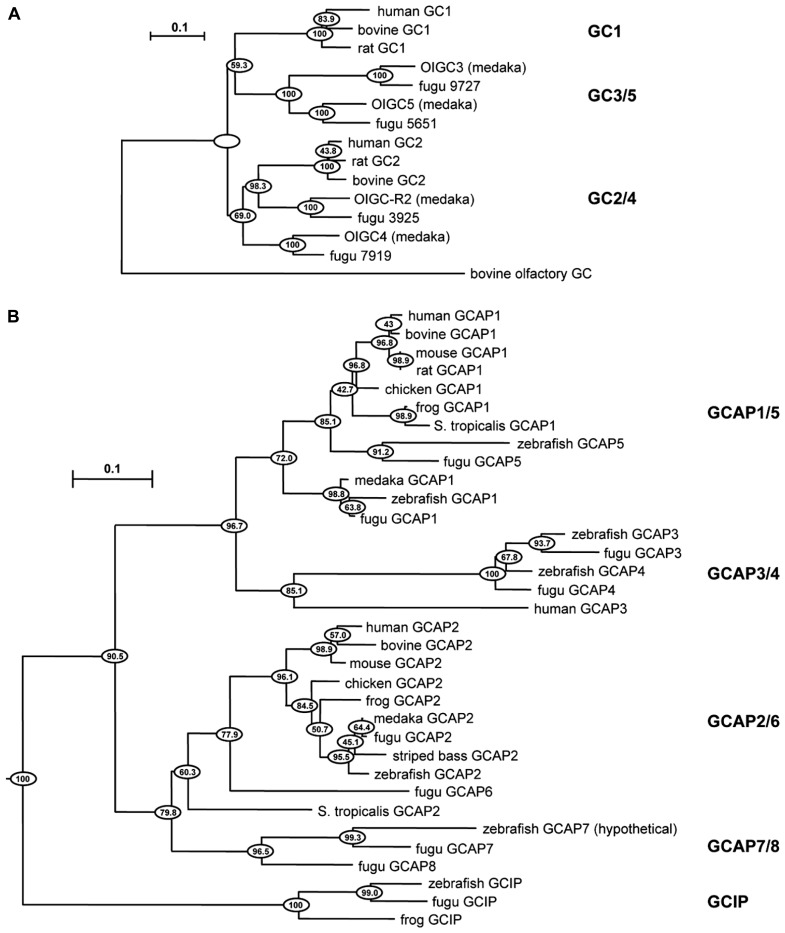
**Evolutionary relationships of GC and GCAP isoforms in vertebrates.** Horizontal branch lengths for GC **(A)** and GCAP **(B)** isoforms are proportional to genetic distances. The scale bar indicates 0.1 amino acid substitutions per site. Clustering percentages at the nodes derived from 1000 bootstrap resamplings. Modified from Imanishi et al. (2004) with permission from Springer.

Negative feedback onto cGMP synthesis provided by a Ca^2+^ sensing GCAP is likely to have already been in place in very ancient ciliary photoreceptors (reviewed in Lamb, 2013). Two rounds of whole-genome duplications predating the origin of vertebrates could have generated multiple isoforms. A third genome duplication in fish could explain why they have at least four GCs and eight GCAPs (Imanishi et al., 2004), although it is not clear whether all are expressed in photoreceptors (**Figure 1**). The existence of multiple isoforms of GC and GCAP provided a substrate for natural selection to optimize the photon response for particular visual ecologies. Where an overly complex system of GCs and GCAPs was not needed, components were eventually lost. For example in mouse, three frame deletions degraded the GUCA1C gene for GCAP3 into a pseudogene (Imanishi et al., 2002). But multiple GCs and GCAPs were retained in some species and were possibly even supplemented with GC inhibitory protein (GCIP; **Figure 1**), that inhibits GC at high Ca^2+^ but does not stimulate it at low Ca^2+^ (Li et al., 1998), and unrelated S100 proteins that stimulate GC1 at high Ca^2+^ (reviewed by Sharma et al., 2014), to tune GC synthesis in order to meet more challenging demands on vision. Over 400 million years of evolution, GCAP1 and GCAP2 genes are ubiquitously preserved in mammals in a tail-to-tail array (Surguchov et al., 1997). In human, there are two isoforms of GCs, RetGC1, and RetGC2 (Lowe et al., 1995), and two isoforms of GCAPs (GCAP1 and GCAP2) in rods (Dizhoor et al., 1994; Palczewski et al., 1994), while at least some cones express an additional GCAP3 (Haeseleer et al., 1999) and possibly only one type of GC (Karan et al., 2010). Three types of GCs and six types of GCAPs are expressed in zebrafish UV cones (Imanishi et al., 2002, 2004; Rätscho et al., 2009), although it remains to be seen whether all are present in the outer segment (**Table 1**). GCs and GCAPs have responsibilities in the inner segment (reviewed in Karan et al., 2010) and synapse of photoreceptors (reviewed by Schmitz, 2014), both of which constitute separate Ca^2+^ compartments. Here, we review the progress that has been made in understanding why so many types of GCs and GCAPs are involved in phototransduction. First, we will consider a relatively simple system that utilizes predominantly two components, GC1 and GCAP1. The impact of genetically deleting the GCAP revealed its role in shaping the response to light. Next, we will describe how this approach was extended to a more complex system that utilizes two additional components. Finally, an extrapolation will be made to other systems in which genetic studies have not yet been carried out or are still in early stages.

**Table 1:** Distribution of GCs and GCAPs in photoreceptors of mouse, carp, and zebrafish.

	Mouse		Carp		Zebrafish			
	Rod, μM	Cone	Rod, μM	Cone, μM	Rod	UV cone	B cone	R/G Double cone
GC1	3.2–5.8	+	3.9	–	+	+	–	–
GC2	0.8–1.4	–	0.3	–	+	+	–	–
GC3	–	–	–	–	–	+	+	+
GC5	–	–	–	72	?	?	?	?
GCPA1	+*	+	0.84	–	+	+	–	–
GCPA2	+*	–	1.8	–	+	+	–	–
GCPA3	–	–	–	33	–	+	+	+
GCPA4	–	–	–	–	–	+	+	+
GCPA5	–	–	–	–	–	+	+	+
GCPA7	–	–	–	–	–	+	+	+

Results on mouse and carp refer to photoreceptor outer segments, while results on zebrafish were obtained from an analysis of mRNA so the proteins may not necessarily localize to the outer segments. No distinctions are made for cones of different spectral type in mouse or carp. Where the concentrations have not been determined, positive expression is marked with (+), lack of expression is marked with (-), unknown status is marked with (?). Expression of GC2 and GCAP2 is negligible in mouse cones [neither GC2 nor GCAP2 is expressed in the all-cone retina of Nrl^-/-^ mouse Xu et al., 2013]. Expression of GCAPs 4 and 7 is presumed to be absent in carp retina based on exceedingly low mRNA levels. Expression of GCAP4 is moderate in zebrafish UV cones. GCAP7 is present in zebrafish cones but at very low levels. ^*Total concentration of GCAPs 1 and 2 in mouse rods is likely between 3 and 9 μM. From Howes et al., 1998; Imanishi et al., 2002,^2004^; Baehr et al., 2007; Rätscho et al., 2009; Takemoto et al., 2009; Peshenko et al., 2011; Scholten and Koch, 2011.

## A SIMPLE SYSTEM IN MOUSE CONES

Mouse cones use a simple system for cGMP synthesis with a single type of GC, GC1, coupled almost exclusively to GCAP1 (**Table 1**). To understand how the Ca^2+^ feedback onto cGMP synthesis influences the cone photoresponse, it is useful to simplify the system further by genetic engineering. The tacit assumption is that the genetic manipulation “cleanly” removes a targeted protein(s) in an otherwise undisturbed system. Caution is necessary because full verification is impossible and there may be unpredictable consequences. As an illustration, knockout of GC1 in mice interferes with the expression of many cone phototransduction proteins, including transducin, PDE, GCAP1, and arrestin, and the cones eventually degenerate. Surprisingly, small cone ERG responses to light were detected at 1 month of age (Yang et al., 1999), but they soon vanished over the next few weeks as the cones degenerated (Yang et al., 1999; Baehr et al., 2007).

**FIGURE 2:**
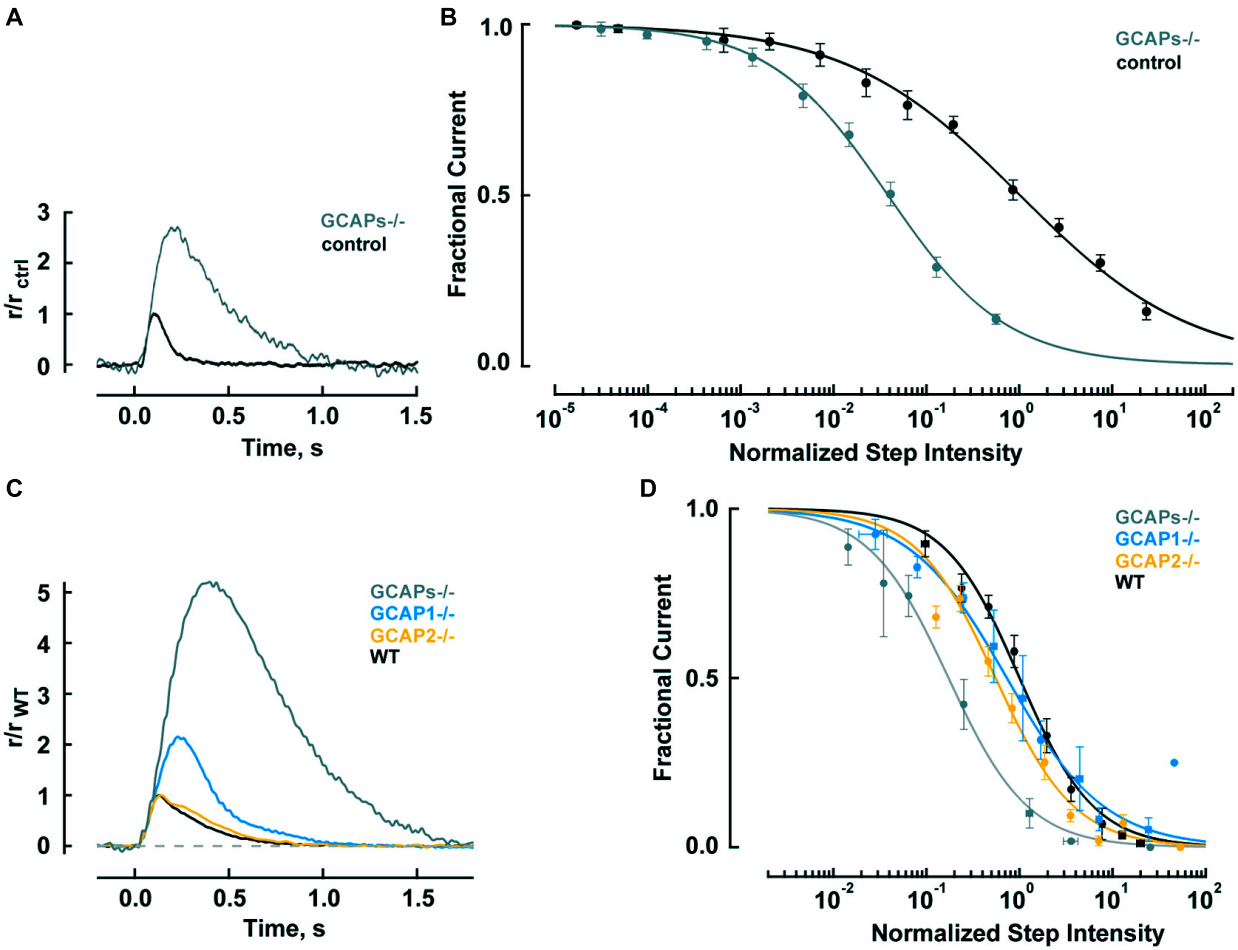
**GCAPs shape the photon responses and set the operating ranges of cones and rods. (A)** Enlarged, slowed photon response of cones from mice lacking the α-subunit of rod transducin, to render rods unresponsive to light, and both GCAPs (Gnat1^-/-^, GCAPs^-/-^). Control response is from Gnat1^-/-^ cones. Mean responses to flashes given at time zero are normalized by the peak amplitude of the control cone response, 0.01 pA. For reasons that are not known, cone response kinetics of this study were slower than those reported by Cao et al. (2014) in **Table 2**. **(B)** Shift in the operating range of GCAPs^-/-^ cones to lower intensities. Fractional current is that present 2 s after light onset. Step intensities are normalized to the mean intensity suppressing half of the dark current for controls, *I*_1/2_ = 94,300 hυ μm^-2^ s^-1^. Continuous lines are fits with the Hill equation for GCAPs^-/-^ and control cones with Hill coefficients of 0.78 and 0.49, respectively. **(A,B)** were adapted from Sakurai et al. (2011) with permission from Society for Neuroscience. **(C)** Mean single photon responses of WT, GCAP1^-/-^, GCAP2^-/-^, and GCAPs^-/-^ rods with amplitudes of 0.47, 1.02, 0.43, and 2.34 pA, respectively, before normalization, includes results from Makino et al. (2008, 2012b). **(D)** Progressive shift in the rod operating range to lower intensities as one or both GCAPs are knocked out. Fractional current is that remaining after 10 s of illumination. The mean I_1/2_ for WT rods was 480 hυ μm^-2^ s^-1^. Traces show fits with Hill equation with Hill coefficients of: 1.1, 0.8, 1.0, and 1.0 for WT, GCAP1^-/-^, GCAP2^-/-^ and GCAPs^-/-^. **(B,D)** Error bars show SEM.

Genetic deletion of GCAPs (GCAPs^-/-^) may be a more specific experimental perturbation and cones retain good health. **Figure 2A** shows that the single photon response of a GCAPs^-/-^ cone rises normally, but continues to do so for twice as long to finally reach a peak after 220 ms that is 2.7-fold higher than normal (Sakurai et al., 2011). Integration time, calculated as the integral of the response divided by response amplitude, increases by 2.3-fold. Loading a WT cone with a Ca^2+^ buffer to delay the onset of Ca^2+^-dependent feedback also prolongs its photon response but such treatment does not affect the flash response of a GCAPs^-/-^ cone because GCAP1, the main mediator of the Ca^2+^-dependent negative feedback onto the phototransduction cascade that dynamically shapes the photon response in mouse cones, is not present.

Since responses to dim steady light summate the responses to individual photons, the large, slow photon responses in GCAPs^-/-^ cones should shift the response-intensity relation for steps of light to light intensities (2.7 × 2.3) = 6.2 times lower. There is such a shift for very weak responses, however, GCAPs^-/-^ cones are about 20-fold more sensitive than wild type (WT) cones at the half maximal response level (**Figure 2B**; Sakurai et al., 2011). The extra shift suggests that the feedback regulation provided by GCAP1 becomes more vigorous when extended exposures to brighter light cause Ca^2+^ to drop lower than the minimum reached during a single photon response. The steeper GCAPs^-/-^ response-intensity relation for steps of light shows that GCAP1 normally enables cones to operate over a wider range of intensities.

Thus GCAP1 restricts the growth and quickens the recovery of the single photon response in mouse cones, making them less sensitive but improving their temporal resolution. GCAP1 also extends the operating range of cones by preventing saturation when cones are exposed to bright, steady illumination.

## GREATER COMPLEXITY IN MOUSE RODS

In mouse rod outer segments, two GCs coupled to two GCAPs are responsible for cGMP synthesis (**Table 1**). As with mouse cones, a genetic approach is useful for trying to understand how the system works, but with rods, it is necessary to knock out components individually and in combinations to isolate the physiological function of each component (Yang et al., 1999; Baehr et al., 2007; Makino et al., 2008, 2012b). Mutant mice induced to express transgenes for either GCAP1 or GCAP2 on the GCAPs^-/-^ background are also informative (Mendez et al., 2001; Howes et al., 2002; Pennesi et al., 2003).

Rods are tasked with counting single photons and are therefore designed to be far more sensitive than cones. Major modifications in rods include slowing of the shutoff and recovery phases of the cascade to grant the photon response time to rise to a large size (reviewed in Luo et al., 2008; Gross and Wensel, 2011; Korenbrot, 2012). In mouse, the rod photon response peaks after 140 ms at an amplitude of 0.6 pA, compared to 80 ms and 0.012 pA in cones (**Table 2**). Negative feedback provided by GCAPs is a key factor. The feedback during the single photon response is somewhat more powerful in mouse rods than in mouse cones (**Figure 2**). In the absence of both GCAPs the rod response rises for three times longer than normal to reach an amplitude that is four times larger (Mendez et al., 2001; Burns et al., 2002). The shift in sensitivity for the GCAPs^-/-^ rod is to flashes 6–8 times dimmer compared to WT rods, greater than the shift of 2–3 times for cones after knockout of both GCAPs (**Figure 2A**). When GCAP2 alone is missing (GCAP2^-/-^), the size of the single photon response in rods is normal and the only change is a slightly slower recovery (Makino et al., 2008). Knockout of GCAP1 alone in rods (GCAP1^-/-^) produces a single photon response roughly twice the normal size (Makino et al., 2012b). So between the two GCAPs, GCAP1 must be the primary determinant for setting single photon response amplitude. GCAP2 assumes greater importance with stronger flashes (Mendez et al., 2001; Makino et al., 2008). Bright flashes saturate the rod response; the brighter the flash, the longer it takes for the response to emerge from saturation. GCAP2 acts to reduce the saturation time to an extent roughly equivalent to lowering flash strength 3.8-fold (Makino et al., 2008). Loading WT rods with a Ca^2+^ buffer increases the size of the photon response and alters its kinetics but has no effect on GCAPs^-/-^ rods, indicating that as with cones, no other major Ca^2+^ feedback besides GC/GCAP comes into play during the flash response (Burns et al., 2002).

**Table 2:** Flash response kinetics and relative sensitivity.

	Rod/cone	a, pA	*t*_p_, ms	*t*_i_, ms	*i*_1/2_, hν μm^-2^
Monkey	Rod	0.7	190	275	16
Mouse	Rod	0.6	142	269	56
Chipmunk	Rod	0.5	116	183	153
Monkey	Cone	0.035	48	49	1744
Mouse	Cone	0.012	82	87	5330
Squirrel	Cone	0.005	29	37	16115
Salamander	S cone	0.3	459	1209	235
	L cone	0.03	155	325	1409

**T* = 35–38°C for mammalian cells, *T* = 20–22°C for salamander cells. Amplitude (a), time to peak (*t*_p_) and integration time (*t*_i_) are for the single photon response, typically obtained by analysis of dim flash responses in the linear range. Values for *i*_1/2_ give the flash strengths at λ_max_ that elicit a half maximal response and are thus inversely proportional to relative sensitivity. Flash responses of different spectral types of mammalian cones do not vary so they were averaged: L, M, S cones for primates; M, S for mouse and squirrel. From Baylor et al., 1984; Schnapf et al., 1990; Perry and McNaughton, 1991; Zhang et al., 2003; Isayama et al., 2006,^2014^; Cao et al., 2014. The single photon response amplitude from chipmunk rods studied in Zhang et al. (2003) was provided by T. Kraft, personal communication. Results from mouse were selected from the most recent reports where all parameters were available and in which recording methods were similar: Yang et al., 1999; Mendez et al., 2001; Makino et al., 2004,2012a,2012b; Luo and Yau, 2005; Tsang et al., 2006; Krispel et al., 2007; Woodruff et al., 2007; Herrmann et al., 2010.
*

**FIGURE 3:**
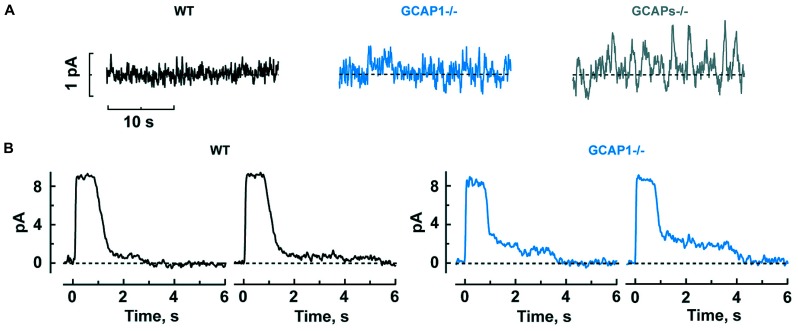
**GCAP1 reduces the cGMP fluctuations underlying the dark noise and response tails after exposure to bright light in rods. (A)** Progressive increase in dark noise after GCAP1 and GCAPs knockout, respectively. **(B)** More prominent photocurrent tails that prolong the recovery after bright light in GCAP1^-/-^ rods. The staircased recovery reflects the stochastic shutoff of aberrant single photon responses generated during the light exposure. Traces are from single trials, where a bright flash was turned on at time zero. Reproduced from Makino et al. (2012b).

It might seem that enlarged single photon responses should improve the ability of GCAPs^-/-^ and GCAP1^-/-^ rods to count photons. But when GCAPs are not present, there is a 40-fold greater variance of the dark noise (Burns et al., 2002), which would instead diminish the accuracy of detecting photons. Phototransduction noise stems from two sources: discrete noise due to the thermal activations of rhodopsin (Baylor et al., 1980) and continuous noise due to spontaneous PDE activation (Rieke and Baylor, 1996). GCAPs counteract the cGMP depletion caused by both components (Burns et al., 2002). GCAP1^-/-^ rods are also noisy, albeit somewhat less so, whereas GCAP2^-/-^ rods appear to be normal, indicating that GCAP1 is more important for dampening the noise although GCAP2 likely comes into play when GCAP1 is not present (**Figure 3A**).

In steady light, given that stimulation of GC activity by GCAPs increases with light intensity as Ca^2+^ is driven down to lower and lower levels, one might expect GCAPs to extend the operating range of rods by keeping them out of saturation, just as GCAP1 does in cones (**Figure 2B**). But that does not happen; the relation between fractional current and light intensity does not fall more gradually in WT rods compared to GCAPs^-/-^ rods (**Figure 2D**). One explanation might be that something happens at higher light intensities, such as the summation of long lasting, aberrant photon responses (see below), which undermines the efforts of GCAPs in their attempt to help rods evade saturation. Interestingly, knockout of GCAP1^-/-^ does extend the operating range of rods, perhaps compensatory overexpression of GCAP2 boosts the maximal GC activity and the stimulation of GC activity is concentrated at higher intensities (Makino et al., 2012b).

Mammalian rods possess an odd characteristic: about once per several hundred events, rods generate an aberrant single photon response that climbs to an amplitude nearly twofold larger than normal and then remains at that amplitude for an average of 3–6 s, before recovering. In individual trials, the duration is unpredictable and some aberrant responses last for tens of seconds (Baylor et al., 1984; Chen et al., 1995; Kraft and Schnapf, 1998). Apparently, rhodopsin excitations are not always shut off properly by phosphorylation and arrestin binding (Chen et al., 1995, 1999). The aberrant response is enhanced in the absence of GCAP1, to a greater extent than the normal single photon response (Makino et al., 2012b). Even though aberrant responses are relatively rare, they have a significant physiological impact, because they produce a “tail” that delays the recovery after bright flashes. Tails are especially prominent when GCAP1 is missing (**Figure 3B**).

**FIGURE 4:**
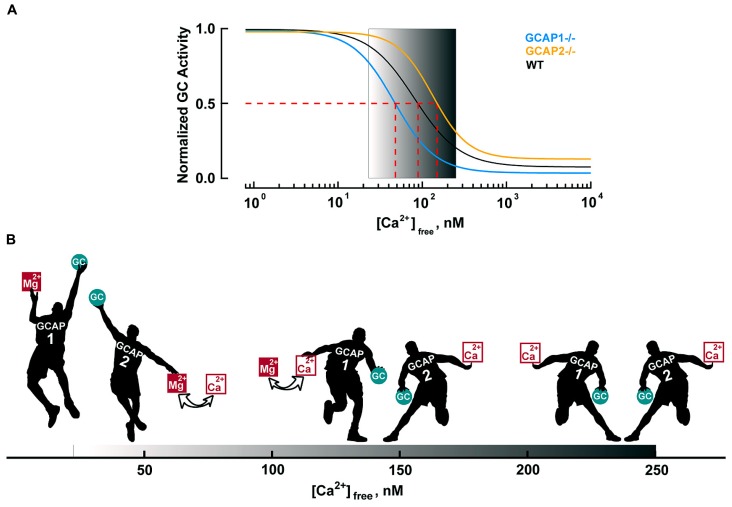
**Ca^**2+**^-dependent regulation of GC activity in mouse rods. (A)** Ca^2+^ dependence of GC activity for each GCAP isoform and for the mixture in mouse rods. GC activities are normalized to their respective maxima and follow the Hill equation: *A* = (A_max_–A_min_)/(1 + ([Ca^2+^]/K_1/2_)^nH^)+ A_min_ with nH values of 1.8, 1.6, and 2.1 for WT, GCAP1^-/-^ and GCAP2^-/-^, respectively. The physiological range of internal free Ca^2+^ is 23 nM in the light to 250 nM in the dark, as demarcated by the gradient background. Modified from Makino et al. (2008, 2012b).** (B)** Recruitment model. The physiological free Ca^2+^ concentration range shown by the black progression at the bottom of the figure. In darkness (right), nearly all of the GCAP1 and 2 “players” bind Ca^2+^ and suppress GC activity (only a single representative player of each type is shown). Soon after illumination, both GCAPs respond to the light-induced Ca^2+^ concentration decrease. The disparity in Ca^2+^ affinity causes many more GCAP1 players to release their bound Ca^2+^ than GCAP2 players. In that sense, GCAP1 is the “first responder”. Mg^2+^ replaces the released Ca^2+^ on GCAP, prompting it to raise GC activity (Peshenko and Dizhoor, 2004). As intracellular Ca^2+^ falls further, the rest of the GCAP2 players exchange Mg^2+^ for bound Ca^2+^ and they join the GCAP1 players in lifting GC activity. Very few GCAP2s exchange Ca^2+^ for Mg^2+^ during the single photon response, but with bright light, all GCAP1 and GCAP2 players get involved (far left). During the recovery of the response to light, cyclic nucleotide gated (CNG) channels reopen to allow Ca^2+^ back in. GCAP2 players regain their Ca^2+^ first and begin to restrain GC. Gradually, as intracellular Ca^2+^ levels return to baseline, GCAP1 players also regain Ca^2+^ and turn down GC activity.

The basis for the different roles of GCAP1 and GCAP2 lies in their Ca^2+^ sensitivities. GCAP1 has a lower affinity for Ca^2+^ than GCAP2 (**Figure 4A**). The K_1/2_ for Ca^2+^ of GCAP1 in the mouse rod is 130–140 nM, while that of GCAP2 is 50–60 nM (Makino et al., 2008; Peshenko et al., 2011). Levels of free Ca^2+^ inside a mouse rod range from 250 nM in darkness to 23 nM in saturating light (**Table 3**). So during the initial fall in Ca^2+^ incurred during the photon response, many more GCAP1 molecules release their Ca^2+^ than GCAP2. The majority of GCAP2 molecules only release their bound Ca^2+^ when intracellular Ca^2+^ plummets, during the saturating response to very bright light. A model illustrating the actions of GCAP1 and GCAP2 is shown in **Figure 4B**. A similar “relay” model was proposed earlier (Koch and Dell’Orco, 2013) but we here prefer the term “recruitment” model to avoid conveying inadvertently the impression that GCAP2 stimulation substitutes for GCAP1 stimulation at low Ca^2+^. “Recruitment” emphasizes that GCAP2 adds to stimulation of GC by GCAP1.

**Table 3:** Dynamics of intracellular free Ca^**2+**^ in the outer segments of rods and cones.

Type	Time constants, ms	[Ca^2+^]_dark_, nM	[Ca^2+^]_light_, nM	[Ca^2+^]_dark_/[Ca^2+^]_light_
Mouse rod	154, 540	250	23	11
Lizard rod	580, 5450	554	50	11
Salamander rod	260, 2200	410–670	30	14–22
Salamander cone	43, 640	410	5	82

*Intracellular free Ca^2+^ declines after sudden closure of CNG channels by bright light as the sum of two exponential functions. The two time constants listed are from measurements of fluorescent Ca^2+^ indicators. Electrophysiological measurements of Ca^2+^ exchange are not included because they often do not resolve the second, slower component. *T* = 37°C for mouse rod, and *T* = 16–23°C for lizard and salamander cells. From Woodruff et al., 2002; Gray-Keller and Detwiler, 1994; Lagnado et al., 1992, Sampath et al., 1998,^1999^.*

**FIGURE 5:**
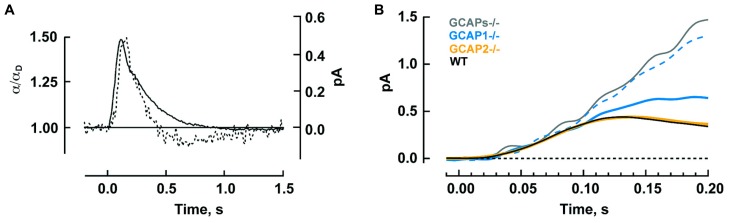
**Timeline for GCAP regulated GC activity during the photon response in mouse rods. (A)** The calculated time course of GC activity α normalized to the basal value in darkness (broken trace) overlaid on the single photon response (solid trace). The two curves rise with similar slopes to reach their peaks 40 ms apart. Thereafter, GC activity declines sharply. Reproduced from Burns et al. (2002) with permission from Elsevier. **(B)** Divergent rising phases of the photon responses of WT rods and rods lacking either or both GCAPs. The GCAPs^-/-^ response separates from the WT response at 70–80 ms after the flash. Separation of the larger response of those GCAP1^-/-^ rods presumed to have little GCAP2 overexpression (dashed blue trace) is next, at 90–100 ms, followed by the smaller response of those GCAP1^-/-^ rods presumed to have the most GCAP2 overexpression (solid blue trace) at 100–110 ms. The response of GCAP2^-/-^ rods overlays the WT until well after the peak. Includes results from Makino et al. (2008), 2012b.

The timing of Ca^2+^ feedback onto GC activity during the photon response is shown in **Figure 5A**. GC activity ascends at the same rate as the electrical response with a ~40 ms delay, then declines sharply and falls briefly to a level less than the dark value. GCAP1’s lower affinity to Ca^2+^ makes it the logical “first responder” to the initial decrease in Ca^2+^ (**Figure 4**). Although it could have been the case that GCAP2 responds later because it has a slower release of Ca^2+^ than GCAP1 after a sudden drop in intracellular Ca^2+^, it will be argued below that both GCAPs are rapid Ca^2+^ sensors. **Figure 5B** shows the rising phases of the photon responses of mouse rods lacking GCAP1, GCAP2, or both. The GCAPs^-/-^ response is the first to diverge at ~80 ms after the flash, while the GCAP2^-/-^ response is the last to diverge. Based on this pattern, it may be inferred that GCAP1 senses the fall in Ca^2+^ and begins to stimulate GC by 80 ms post-flash. GCAP1^-/-^ rods are highly variable in their response waveforms apparently because they upregulate GCAP2 expression and the degree of compensation differs from rod to rod. Rods with the least compensation show response divergence from the WT response nearly as early as the GCAPs^-/-^ response. Accordingly, GCAP2 must respond rapidly to the initial fall in Ca^2+^. At normal expression levels, the paltry number of GCAP2 molecules responding to the initial fall in Ca^2+^ is unable to make its presence felt. But the mutant rods show that given enough GCAP2, its feedback onto GC does restrain the early rising phase of the response.

As one might expect, knockout of both GCs in rods eliminates the dark current, sabotaging their capacity for phototransduction (Baehr et al., 2007). Deleting GC2 alone seems to have little effect on the dark current or on flash response kinetics and sensitivity in recordings from individual mouse rods (Baehr et al., 2007). In contrast, GC1 deletion compromises the expression of both GCAPs revealing differences between the duties of the two GCs not directly related to phototransduction in the outer segment. There is an increase in flash sensitivity and a delayed time to peak of the photon response (Yang et al., 1999; Baehr et al., 2007), as predicted from the reduced levels of GCAPs (Mendez et al., 2001). Paradoxically, elimination of GC1 also accelerated the rod response recovery (Yang et al., 1999).

In summary, the functional significance to phototransduction of expressing two types of GCs in rods is not yet clear. For GCAPs, there is a division of labor. GCAP1 helps to achieve the proper balance of keeping noise to a minimum while restricting photon response size to optimize the range of flash strengths and light intensities over which the rod operates. Contrary to the situation in cones, the shutoffs of photoexcited visual pigment and activated transducin are slow in rods to allow the photon response to grow. The slow shutoffs pose a problem with bright flashes because the time that the rod stays in saturation gets prolonged. GCAP2 addresses that problem by boosting GC activity to bring the rod out of saturation sooner.

## TUNING Ca^**2+**^ FEEDBACK WITH ADDITIONAL GCs AND GCAPs BETWEEN DIFFERENT ANIMAL SPECIES

While it is generally true that the photon response of rods is larger and slower than that of cones, the response can vary across rods and across cones of different species. The preceding sections described how Ca^2+^ feedback onto cGMP synthesis reduces the size of the single photon response and quickens its kinetics in mouse rods and cones. The extent to which that occurs is dependent upon the selective expressions of GC(s) and GCAP(s). Therefore, variation that exists in the sensitivity and flash response kinetics of rods and cones from other vertebrates is likely to arise at least in part from differences in their cGMP synthetic machinery. This section will present a few examples of the variation between species and lay out three mechanisms by which cGMP production could be altered to tune the photon response: by augmenting the amounts of GC and GCAP expressed, by a change in intracellular Ca^2+^ dynamics or by a switch in the types of GCs or GCAPs expressed.

**FIGURE 6:**
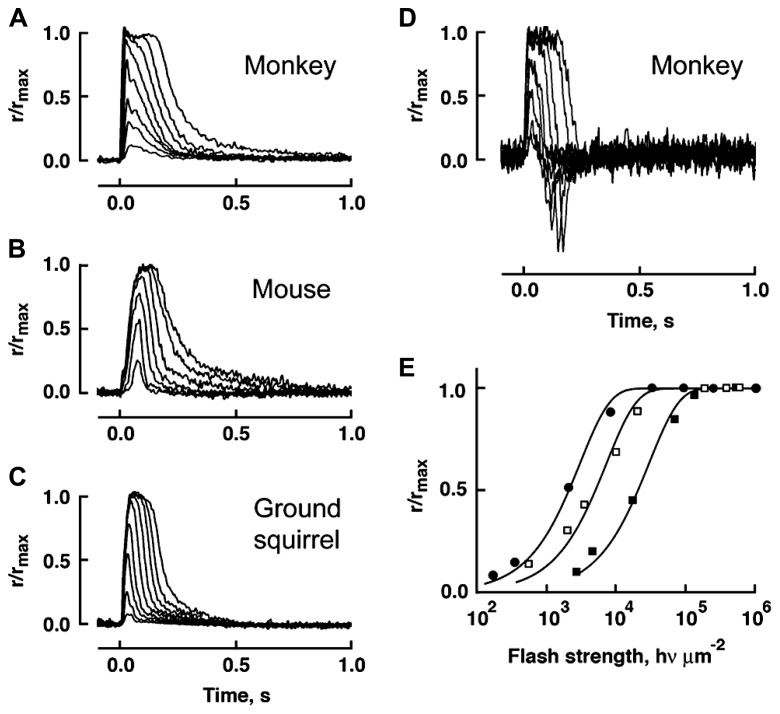
**Variation in flash response kinetics and sensitivity across mammalian cones for monkey (**A**), mouse **(B)** and squirrel **(C)**.** A monkey cone with an undershoot is shown in **D**. **(E)** The stimulus-response relations for the cones in **A–C** showing the order of relative flash sensitivity. The monkey cone (circles) is more sensitive than the mouse cone (open squares), which is more sensitive than the squirrel cone (filled squares). Modified with permission from Cao et al. (2014).

In comparison to mouse rods, primate rods have a single photon response that peaks later whereas the response of chipmunk rods peaks and recovers earlier (**Table 2**). In general, the single photon response of cones is far smaller and peaks much sooner than the response of rods in the same animal. Across species, ground squirrel cones generate smaller, faster photon responses than mouse cones (**Figure 6**; **Table 2**). At present, primate cones are something of an enigma. Their single photon response kinetics are faster than those of mouse yet the single photon response is larger and relative sensitivity is greater. In initial studies on isolated primate retina, cone flash responses exhibited a prominent and reproducible undershoot to the recovery (Baylor et al., 1987; Schnapf et al., 1987; Schnapf et al., 1990). Under the same conditions, cones of other mammals did not generally have undershoots, with the possible exception of chipmunk cones (Zhang et al., 2003). However, in a later study on primate cones, undershoots were absent or only seen occasionally (**Figure 6**) and kinetics inferred for cones in the intact human eye lack the undershoot (van Hateren and Lamb, 2006). Although flash response kinetics are invariant across cone spectral type in mammal (e.g., Baylor et al., 1987 and Cao et al., 2014), there are distinct differences across cone type in fish and amphibians where faster time to peak corresponds with reduced sensitivity (Perry and McNaughton, 1991; Miller and Korenbrot, 1993; Isayama et al., 2006, 2014). After a partial bleach of the visual pigment content, flash responses of salamander L cones develop an undershoot but flash responses of S cones do not (cf. Figures 4–6 of Isayama et al., 2006).

It has been proposed that an enhanced expression of GC in cones accounts, in part, for their faster flash response kinetics and their ability to signal over an enormous range of light intensities (Takemoto et al., 2009). Expression of GC is 17 times higher in carp cones than in rods (**Table 1**) and the basal rate of cGMP synthesis in cones is an order of magnitude higher. The differential appears to be lower in salamander to account for a basal rate that is only ~3-fold higher in their cones compared to their rods (Cornwall and Fain, 1994; Cornwall et al., 1995). But despite the higher basal activity in cones, the fold change in stimulation of GC activity in the light is greater in rods than in cones for both carp and salamander. GC will function in the absence of GCAP, so a lower fold change in GC synthesis at low Ca^2+^ could be achieved by reducing the ratio of GCAP to GC. That turns out not to be the case in carp, indicating that there are quantitative differences in the way that the cGMP synthetic machineries of rods and cones respond to low Ca^2+^ and/or differences in their Ca^2+^ dynamics (Takemoto et al., 2009).

Dissimilarities in the operation of the same cGMP synthetic machinery may exist across photoreceptors because of how they handle Ca^2+^. Ca^2+^ dynamics in the outer segment depend upon the rates of Ca^2+^ entry through the CNG channel and removal by a Na^+^/K^+^, Ca^2+^ exchanger, as well as Ca^2+^ buffering and outer segment surface to volume ratio. In cones, the fraction of circulating current into the outer segment carried by Ca^2+^ is roughly twice that in rods, due to a greater permeability of the cone CNG channel for Ca^2+^ (Nakatani and Yau, 1988; Perry and McNaughton, 1991; Ohyama et al., 2000). It is not clear whether the cone exchanger operates at a faster rate than the rod exchanger, but surface to volume is greater in cone outer segments and their time constant for Ca^2+^ extrusion is faster (Sampath et al., 1999; Paillart et al., 2007). As a result, [Ca^2+^] changes more rapidly and the fold change in Ca^2+^ levels is bigger in cones than in rods (**Table 3**). In salamander, a faster rate of Ca^2+^ extrusion in L cones than in S cones may contribute to faster response kinetics of L cones (Perry and McNaughton, 1991). In both salamander rods and cones, the dark level of Ca^2+^ is higher than in mouse rods, and in salamander cone, the light induced fall in Ca^2+^ is to 5 nM, a lower level than that in mouse rod, 23 nM (Sampath et al., 1999; Woodruff et al., 2002). For feedback on cGMP synthesis to operate over the full range of Ca^2+^ concentrations, the choice of GCAP(s) expressed will need to be based in part on Ca^2+^ affinity.

**FIGURE 7:**
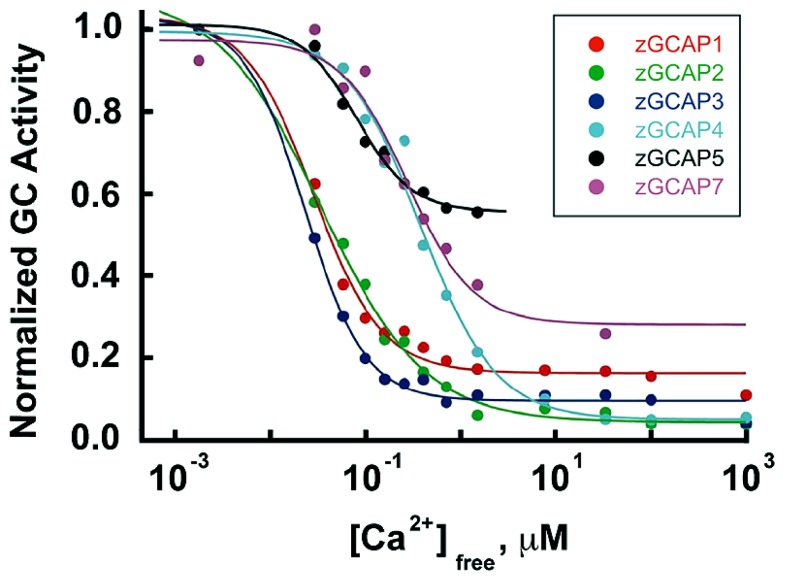
**Ca^**2**+^ dependence and extent of GC activation by zebrafish GCAPs.** Reconstituted zebrafish GCAPs were assayed *in vitro* against bovine rod outer segment membranes that were stripped of native GCAPs but contained a mixture of GC1 and GC2, with GC1 predominating. Activity is plotted relative to the maximum rate for each isoform. *K*_1/2_ values are 30, 35, 25, 520, 440 and 180 nM, and the fold activation values are 3, 10, 6, 13, 1.5, and 2 for zGCAP1-5 and 7, respectively. From Scholten and Koch (2011).

The occurrence of isoforms of GC and GCAP allows for adjustments to be made in their biochemical properties: K_1__/2_ for Ca^2+^, cooperativity for Ca^2+^ binding, maximal and basal rates of cGMP synthetic activity. Further flexibility stems from having maximal stimulation of GC depend on the pairing of a GC with specific GCAP isoforms. The unstimulated rate of cGMP synthesis by mouse GC1 is nearly 10-fold less than that of GC2. But stimulation of GC1 activity by GCAP1 and GCAP2 is 28-fold and 13-fold, respectively, whereas for GC2 activity, it is only sixfold and fivefold, respectively (Peshenko et al., 2011). In some cases, the effects of GCAPs on GC are species dependent. Zebrafish GCAP5 will stimulate carp rod GCs severalfold at low Ca^2+^ concentration but has little effect on carp cone GC (Takemoto et al., 2009). The 6 zebrafish GCAPs differ in the degree to which they will stimulate bovine rod GC activity, ranging from 1.5- to 13-fold (**Figure 7**), but stimulation of zebrafish GC3 by GCAPs 3–5 and 7 is more uniform at ~2-fold (Fries et al., 2013). Carp GCAP1 stimulates carp GC preparations consisting mostly of GC1 to a greater extent than GCAP2 (Takemoto et al., 2009), which is just the opposite of the effect of these zebrafish GCAPs on bovine GC (Scholten and Koch, 2011). These comparisons do not carry physiological significance, nonetheless, it is clear that switching GC and GCAP types is important for setting the basal rate of cGMP synthesis at high Ca^2+^, the maximal rate at low Ca^2+^, and the fold activation. In contrast to mouse cones that express GC1 alone, mouse rods add a second GC with a ratio of GC1/GC2 of (4:1) (Peshenko et al., 2011), similar to the (3:1) ratio in bovine rods (Skiba et al., 2013). It should be noted that these recent determinations indicate that the fraction of RetGC2 in mammalian rods is much higher than previously thought (Hwang et al., 2003; Helten et al., 2007).

GCAPs also vary in their K_1/2_ for Ca^2+^ (Hwang et al., 2003; Peshenko et al., 2011), a property that would appear to be important for optimizing feedback onto cGMP synthesis to the light induced fall in Ca^2+^. For example, with K_1/2_ values of 130–140 nM and 50–60 nM (Makino et al., 2008; Peshenko et al., 2011), mouse GCAPs 1 and 2 provide little or no incremental activation of GC below ~10 nM (**Figure 4A**), but intracellular [Ca^2+^] in salamander cones falls to 5 nM upon exposure to bright light. So another GCAP with lower K_1/2_ for Ca^2+^ is needed if cGMP synthetic rate is to increase over the entire Ca^2+^ range. K_1/2_ values for zebrafish GCAPs 1–3 cluster at about 30 nM (**Figure 7**). It remains to be seen whether lower values are possible. Certainly GCAPs, e.g., zGCAPs 4,5, can have K_1__/2_ values higher than those of mouse GCAPs (**Figure 7**), but while such GCAPs may be useful for photoreceptors of lizards and salamanders (**Table 3**), they cause degenerative retinal disease in mammals (reviewed in Behnen et al., 2010). Ca^2+^-dependent activation of GC by GCAPs is cooperative (reviewed in Luo et al., 2008; Gross and Wensel, 2011; Korenbrot, 2012) but so far, there is no evidence for differences in their cooperativity (e.g., Peshenko and Dizhoor, 2004) or in the rapidity of their response to low Ca^2+^ (see above). Given the differences in the composition of their cGMP machineries (**Table 1**), it may be predicted that the physiological properties of zebrafish cones will vary with spectral type.

The selectivity rules for forming GC complexes are not completely understood. The apparent affinity of mouse GC2 is slightly higher for GCAP2 than for GCAP1 *in vitro* (Peshenko et al., 2011), yet in living mouse rods, regulation of RetGC2 is heavily dominated by GCAP2 with very little, if any, contribution from GCAP1 (Olshevskaya et al., 2012). Apparently, the selectivity mechanism taking place *in vivo* is not governed strictly by binding affinity observed *in vitro*. Thus while the Ca^2+^ sensitivity of the regulation is determined by the type of GCAP rather than isozyme of the cyclase (Peshenko et al., 2011), the type of GC expressed could shift the GCAP dominance and the Ca^2+^ sensitivity.

It may be argued that Nature could have given mouse rods a single GCAP having a K_1/2_ for Ca^2+^ that matches the value obtained by their mixture of GCAPs 1 and 2. This path was not chosen, perhaps because the flexibility of a mixture of components allowed for adjustments to be made on a shorter evolutionary time scale to accommodate other changes in the phototransduction cascade, as rods evolved as photoreceptors (Lamb, 2013).

On a much shorter time scale, relevant to the lifetime of an individual, the complexity of a mixture of components leaves open many options for plasticity. The absence of GCAP5 transcripts in zebrafish until 15 dpf (Rätscho et al., 2009), even though at least some cones are present by 3 dpf suggests a changeover in the type of GCAP expressed with age. Since each GC has a rank order of preference for GCAPs with which it will form a complex (Takemoto et al., 2009; Peshenko et al., 2011; Scholten and Koch, 2011; Olshevskaya et al., 2012), relative expression levels of each GCAP may be a factor in determining which GC/GCAP complexes predominate. In zebrafish cones, GCAP3 expression exceeds GC3 expression by an order of magnitude, yet there is no change in optomotor behavior upon knockdown of GCAP3 (Fries et al., 2013). Presumably, GCAP3 is replaced with a GCAP having similar properties. However, it is possible that reduced expression of a different GCAP would result in greater incorporation of GCAP3 with a change in visual function. In mice, knockout of GCAP2 has no effect on GCAP1 expression, but knockout of GCAP1 drives an increase in GCAP2 expression with a greater fractional incorporation of GCAP2 into GC complexes that yields a higher maximal GC activity at low Ca^2+^ (Makino et al., 2012b). It is tempting to speculate that GCAP1 expression may fall under transcriptional control by some conditions. Exposure to bright light reduced PDEα mRNA levels in mice by twofold (Krishnan et al., 2008) and PDEα and PDEβ protein levels in rat by threefold (Hajkova et al., 2010). In rat, there were no changes in GC1 (GC-E) expression; other cGMP synthetic machinery components were not reported. Then unless another GC activity went down, cGMP levels would have risen. GCAPs are sensitive to the [Mg^2+^], which allows them to maintain their cyclase activating conformation in the light and affects the [Ca^2+^] at which they de-activate GC (Peshenko and Dizhoor, 2004). However, evidence so far indicates that internal [Mg^2+^] does not change in rods or in cones upon exposure to light and remains in the vicinity of 1 mM (Chen et al., 2003). So while the potential exists for changes in the choice of GC or GCAP expressed and in their relative levels of expression during development or in response to environmental conditions, the issue of plasticity is at present, unresolved.

Evolution has favored the occurrence of multiple isoforms of GCAPs that vary in their Ca^2+^ affinity and GCs with different activities and preferences for the type of GCAP bound. By expressing selected components and regulating their levels of expression, vertebrates “design” the cGMP synthetic machinery in their photoreceptors in order to adjust their physiological responses to light stimuli. The possibility exists for plasticity during development or on a shorter time scale to meet changing visual demands.

## Conflict of Interest Statement

The authors declare that the research was conducted in the absence of any commercial or financial relationships that could be construed as a potential conflict of interest.
